# Biomarkers of lipid metabolism in gastric cancer: a case control study

**DOI:** 10.1186/s12885-024-12230-9

**Published:** 2024-04-11

**Authors:** Fangxuan Li, Jinli Dou, Lijuan Wei, Shixia Li, Juntian Liu

**Affiliations:** Department of Cancer Prevention, Tianjin Medical University Cancer Institute and Hospital, National Clinical Research Center for Cancer, Tianjin’s Clinical Research Center for Cancer, Key Laboratory of Cancer Prevention and Therapy, Tianjin, China

**Keywords:** Gastric cancer, Lipid metabolism, The risk, Clinicopathologic features, TNM stage

## Abstract

**Background:**

The aim of this study was to explore the correlation between biomarkers of lipid metabolism and gastric cancer.

**Methods:**

1120 gastric cancer patients and 1134 health examiners enrolled in this study. The clinic data and serum lipid level, including Total cholesterol (TC), Triglyceride (TG), Low-density lipoprotein cholesterol (LDL-C) and High-density lipoprotein cholesterol (HDL-C), were collected.

**Results:**

Serum TG and LDL-C levels in patients with gastric cancer were higher than those in the control group. HDL-C levels were lower than the control group (*P* < 0.05). HDL-C and LDL-C were significantly correlated with the risk of gastric cancer. Concentrating on clinicopathological features, increased TG was more frequently in male patients with distal gastric cancer, N0 stage and early TNM stage. Increased TC was more frequently in early T, N and TNM stage. Decreased HDL-C was more common in distal location and low-undifferentiated gastric cancer. LDL-C elevation was more common in distal gastric cancer and early T stage.

**Conclusions:**

The serum lipid level of gastric cancer patients was higher than healthy controls. HDL-C and LDL-C abnormal correlated with gastric cancer risk. However, as the progresses of gastric cancer, poor patient intake, increased tumor consumption, and continuous declining in nutritional status, the levels of TC and TG gradually decreased in advanced gastric cancer.

## Background

Gastric cancer is a common malignant tumor. In the global malignant tumors, the incidence of gastric cancer ranks sixth and the mortality rate ranks the third [1]. It has high incidence in east Asia, especially in China, the incidence and mortality of gastric cancer both rank the second [2]. The common risk factors for gastric cancer include high-salt diet, intake of pickled, smoking, baked and fried food, irregular diet, and Helicobacter pylori (HP) infection [3, 4]. In recent years, with the improvement of people’s living standard, the incidence of abnormal lipid metabolism was significantly increased in China [5]. Dyslipidemia refers to the abnormal metabolism of lipoproteins in the human body, usually manifested as the increase of TG, TC and LDL-C and/or the decrease of HDL-C. It was found that the regulation mechanism of abnormal lipid metabolism caused by dyslipidemia was involved in promoting the occurrence and development of various tumors, including gastric cancer.

At present, there are some studies on the correlation between lipid metabolism and gastric cancer. However, in these studies, the conclusions are still inconsistent. Aleman et al. [3] illustrated that gastric cancer was one of the obesity-related gastrointestinal tumors. Whereas, Kyrgiou et al. [4] found that obesity had a smaller impact on gastric cancer than other obesity-related cancers, such as colon and breast cancer. Hashimoto et al. [6] reported that the risk of gastric cancer for metabolically abnormal obesity patients, in comparing with metabolically healthy obese patients, was increased. Therefore, they suggested that the occurrence of gastric cancer should be more concerned with metabolic abnormalities, rather than obesity itself. In this study, the correlation between serum lipids and gastric cancer, including cancer risk and clinicopathological features, was investigated by a case-control study.

## Methods and materials

### Patients

Retrospective analysis was performed on patients with gastric cancer who were treated from March 2016 to June 2017. Inclusion criteria: (1) Patients with gastric adenocarcinoma confirmed by histopathological examination. (2) Patients with complete clinicopathological data, all the clinicopathological stages were evaluated according to the 8th pTNM staging criteria of gastric cancer in 2016. (3) Patients with complete follow-up data.

Exclusion criteria: (1) Patients received chemoradiotherapy before biopsy. (2) Patients with other chronic diseases or infectious diseases related to hyperlipidemia. (3) with a history of other malignant tumors. (4) with long-term regular application of lipid-regulating drugs. According to the above criteria, 1120 patients with gastric cancer were enrolled, with the median age of 62.0yrs (range from 20 to 84yrs).

We randomly selected health controls by systematic sampling in the same period. Criteria of control group: health examiner who underwent physical examination in the cancer prevention department of our hospital from March 2016 to June 2017. Exclusion criteria: (1) Patients with other chronic diseases or infectious diseases related to hyperlipidemia. (2) with a history of malignant tumors. (3) with long-term regular application of lipid-regulating drugs. 1134 health examiner who were selected as the control group, with the median age of 63.0yrs (range from 23 to 85yrs). The comparison of general conditions in gastric cancer patients and control was shown in Table 1.

**Table 1:** General conditions of gastric cancer group and the control group

Factors		GC ^*^(N)	Control (N)	x^2^	P
n		1120	1134		
Age(years)^#^		60.480 ± 10.334	59.814 ± 10.537	1.514	0.130
Gender	Male	818	822	0.086	0.777
	Female	302	312		
Cancer family history	Yes	81	78	0.108	0.805
	No	1039	1056		
Smoking history	Yes	274	254	1.320	0.271
	No	798	830		
Drinking history	Yes	120	141	1.666	0.210
	No	952	943		
BMI^#^		25.09 ± 3.52	24.88 ± 3.78	1.961	0.172

*GC: gastric cancer group; ^#^Described by *Mean ± SD*, compared by *t test*

This study was approved by the Medical Ethics Committee of Tianjin Medical University Cancer Institute and Hospital.

### Smoking and alcohol consumption

Based on WHO’s standardized recommendations on smoking survey methods, we defined smoking history as those who smoked more than 1 cigarette per day for 6 consecutive or cumulative months. According to China Monitoring Report of Chronic Diseases and Their Risk Factors 2007 Standard, we defined people who drank more than 25 g/d/m (15 g/d/m for women) as having a history of alcohol consumption.

### Blood lipid profile and reference range

5 ml of morning venous blood was collected from the subjects 12 h after fasting, TC, TG, HDL-C and LDL-C were detected by enzyme direct method. Hitachi automatic biochemical analyzer (7600) and the original reagents were used. External quality assessments (EQA) were progressed twice/year, the application and results analysis were according to National Center for Clinical Laboratories of China National Health Commission. Normal level reference range: TG (0.4-1.8mmol/L), TC (2.8-5.2mmol/L), HDL-C(1.07-1.89mmol/L), LDL-C(1.9-3.1mmol/L).

### Statistical analysis

SPSS 20.0 software was used for statistical analysis. Age, TC, TG, HDL-C and LDL-C of gastric cancer patients and control group were all in normal distribution, *Mean ± SD* was used to describe continuous variable, *t* test was used to analyze the differences. The *x*^*2*^ test and *fisher*’s exact test were used to compare the categorical variable. The risk of gastric cancer was analyzed by Logistic regression, and *P* < 0.05(two side) was considered statistically significant.

## Results

### General conditions

There were 818 males and 302 female gastric patients. The general characteristics of gastric cancer and the control group were shown in Table 1. There was no significant differences in age, gender, family history, smoking and drinking history, BMI between the gastric cancer group and the healthy control group (*P* > 0.05).

### Serum lipid level and dyslipidemia rates of gastric cancer patients and control group

**Fig. 1:**
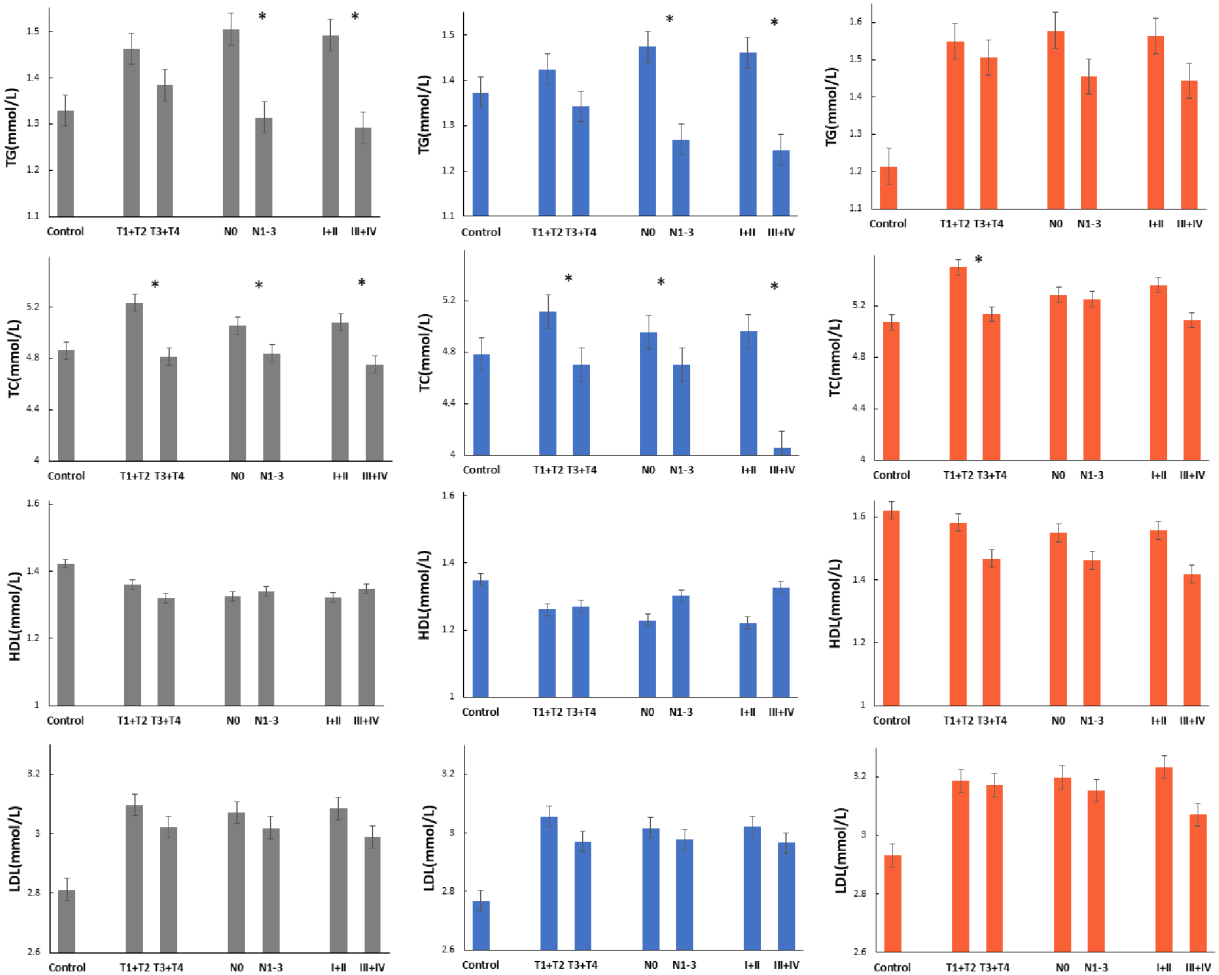
Serum lipid level according to TNM stage of gastric cancer. Gray: total gastric patients, Red: female gastric patients, Blue: male gastric cancer patients, *statistically significant *P* < 0.05

Serum TG and LDL-C levels in patients with gastric cancer were higher than those in the control group. While, HDL-C levels were lower than the control group. The differences were significant statistically both in all patients and females (*P* < 0.05). Beside LDL-C and HDL-C levels, there was no other significant difference between gastric cancer and control group(*P*>0.05) (Table 2; Fig. 1).

**Table 2:** The comparing of serum lipid levels and dyslipidemia rates of gastric cancer group and control group

	Total				Male				Female			
	GC*	Control	t/ x^2^	P	GC	Control	t/ x^2^	P	GC	Control	t/ x^2^	P
TG(mmol/L)^#^	1.409 ± 0.933	1.329 ± 1.055	1.902	0.047	1.367 ± 0.894	1.373 ± 1.085	0.116	0.907	1.522 ± 0.059	1.214 ± 0.055	3.836	< 0.001
TC(mmol/L) ^#^	4.945 ± 1.177	4.862 ± 1.021	1.755	0.076	4.825 ± 1.026	4.782 ± 0.998	0.851	0.395	5.268 ± 1.467	5.072 ± 1.052	1.897	0.058
HDL(mmol/L) ^#^	1.332 ± 0.687	1.423 ± 0.354	3.959	< 0.001	1.267 ± 0.706	1.349 ± 0.337	2.991	0.003	1.508 ± 0.598	1.620 ± 0.319	2.876	0.004
LDL(mmol/L) ^#^	3.045 ± 1.034	2.812 ± 0.854	5.836	< 0.001	2.997 ± 1.052	2.768 ± 0.835	4.884	< 0.001	3.177 ± 0.974	2.930 ± 0.891	3.381	0.001
TG(*n*,%)^&^	225(20.1%)	265(23.4%)	3.562	0.066	149(18.2%)	206(25.1%)	11.328	0.001	76(25.2%)	59(18.9%)	3.501	0.065
TC(*n*,%)^&^	417(37.2%)	407(35.9%)	0.437	0.512	272(33.3%)	269(32.7%)	0.051	0.834	145(48.0%)	138(44.2%)	0.884	0.373
HDL(*n*,%)^&^	179(16.0%)	129(11.4%)	10.135	0.002	138(16.9%)	105(12.8%)	5.452	0.022	41(13.6%)	24(7.7%)	5.613	0.019
LDL(*n*,%)^&^	452(40.4%)	384(33.9%)	10.186	0.001	311(38.0%)	254(30.9%)	9.202	0.003	141(46.7%)	130(41.7%)	1.570	0.223

*GC: gastric cancer group; ^#^ continuous variable compared by *t* test; ^&^ categorical variable compared by *x*^*2*^ test and *fisher*’s exact test

We also compared the dyslipidemia rates between gastric cancer patients and control group, shown in Table 2. According to the reference range of normal blood lipid level, the abnormal rates of HDL-C and LDL-C in patients were significantly higher than those in the control group (*P =* 0.002; *P =* 0.001). Additionally, the abnormal rates of TG in males were significantly higher than those in the control group (*P* = 0.001).

### Correlation analysis between serum lipid level and the gastric cancer risk

Due to the influence of age on lipid levels, age was involved in Logistic regression as a confounder in this study. Multivariate regression analysis showed that LDL-C[OR = 1.570(1.357∼1.815)] was positively correlated with the risk of gastric cancer, HDL-C[OR = 0.616(0.493∼0.771)] was negatively correlated with the gastric cancer risk both for males and females. What’s more, TG [OR = 1.324 (1.074∼1.632)] was positively correlated with the risk of gastric cancer in females (Table 3).

**Table 3:** Logistic regression for serum lipid level and the risk of gastric cancer

Factors	Total		Male		Female	
	Age-OR	M-OR	Age-OR	M-OR	Age-OR	M-OR
TG	1.091(1.002–1.187)	1.061(0.975–1.156)	1.002(0.908–1.106)	0.989(0.892–1.096)	1.438(1.177–1.757)	1.307(1.063–1.605)
*P*	0.045	0.171	0.965	0.833	< 0.001	0.011
TC	1.068(0.990–1.151)	0.892(0.779 − 0.106)	1.040(0.945–1.144)	0.891(0.761 − 0.106)	1.124(0.990–1.276)	0.948(0.744–1.208)
*P*	0.088	0.068	0.425	0.064	0.071	0.668
HDL	0.675(0.553–0.824)	0.612(0.490–0.763)	0.685(0.529–0.886)	0.649(0.491–0.859)	0.601(0.422–0.856)	0.436(0.296–0.642)
*P*	< 0.001	< 0.001	0.004	0.002	0.005	< 0.001
LDL	1.320(1.200-1.454)	1.548(1.341–1.787)	1.372(1.181–1.490)	1.517(1.290–1.784)	1.324(1.111–1.577)	1.493(1.067–2.090)
*P*	< 0.001	< 0.001	< 0.001	< 0.001	0.002	0.019

*OR is described as OR (95%CI).* Age-*OR*: Age adjusted OR. M-*OR*: OR in multivariate analysis

### Association between dyslipidemia rates and clinicopathological features of gastric cancer

In all patients, elevated TG was more frequent in distal gastric cancer, N0 stage patients and I + II stage patients. TC elevation was more common in patients with distal gastric cancer, earlier T stage, N stage and TNM stage. Decreased HDL-C was more common in distal gastric cancer, low and un-differentiated gastric cancer. Elevated LDL-C was common in patients at I + II stage, the differences were statistically significant (all *P* < 0.05) (Table 4).

**Table 4:** Correlation of dyslipidemia rates and clinical characteristics of gastric cancer patients

^*^n, %	Location			Pathologic types			T stage			N stage			TNM stage		
	Proximal	Distal	P	Low/un-	Med/high	P	T_1_ + T_2_	T_3_ + T_4_	P	N_0_	N_1_-N_3_	P	I + II	III + IV	P
*Total*	492	628		870	250		357	763		555	565		658	462	
TG	77(15.7)	148(23.6)	0.001	173(19.9)	52(20.8)	0.788	82(23.0)	143(18.7)	0.109	132(23.8)	93(16.5)	0.003	152(23.1)	73(15.8)	0.003
TC	164(33.3)	253(40.3)	0.018	331(38.0)	86(34.4)	0.300	195(54.6)	222(29.1)	< 0.001	242(43.6)	175(31.0)	< 0.001	288(43.8)	129(27.9)	< 0.001
HDL-C	65(13.2)	114(18.2)	0.027	159(18.3)	20(8.0)	< 0.001	49(13.7)	130(17.0)	0.163	84(15.1)	95(16.8)	0.464	116(17.6)	63(13.6)	0.082
LDL-C	208(42.3)	244(38.9)	0.269	354(40.7)	98(39.2)	0.715	156(43.7)	296(38.8)	0.133	237(42.7)	215(38.1)	0.114	284(43.2)	168(36.4)	0.026
*Male*	396	422		608	210		247	571		390	428		460	358	
TG	58(14.6)	91(21.6)	0.011	109(17.9)	40(19.0)	0.131	55(22.3)	94(16.5)	0.060	86(22.1)	63(14.7)	0.008	100(21.7)	49(13.7)	0.003
TC	131(33.1)	141(33.4)	0.941	204(33.6)	68(32.4)	0.799	125(50.6)	147(25.7)	< 0.001	161(41.3)	111(25.9)	< 0.001	185(40.2)	87(24.3)	< 0.001
HDL-C	52(13.1)	86(20.4)	0.007	121(19.9)	17(8.1)	< 0.001	32(13.0)	106(18.6)	0.053	63(16.2)	75(17.5)	0.641	86(18.7)	52(14.5)	0.132
LDL-C	166(41.9)	145(34.4)	0.031	231(38.0)	80(38.1)	1.000	107(43.3)	204(35.7)	0.042	156(40.0)	155(36.2)	0.280	187(40.7)	124(34.6)	0.082
*Female*	96	206		262	40		110	192		165	137		198	104	
TG	19(19.8)	57(27.7)	0.156	64(24.4)	12(30.0)	0.440	27(24.5)	49(25.5)	0.891	46(27.9)	30(21.9)	0.287	52(26.3)	24(23.1)	0.579
TC	33(34.4)	112(54.4)	0.001	127(48.5)	18(45.0)	0.736	70(63.6)	75(39.1)	< 0.001	81(49.1)	64(46.7)	0.729	103(52.0)	41(40.4)	0.069
HDL-C	13(13.5)	28(13.6)	1.000	38(14.5)	3(7.5)	0.322	17(15.5)	24(12.5)	0.488	21(12.7)	20(14.6)	0.736	30(15.2)	11(10.6)	0.295
LDL-C	42(43.8)	99(48.1)	0.536	123(46.9)	18(45.0)	0.866	49(44.5)	92(47.9)	0.632	81(49.1)	60(43.8)	0.418	97(49.0)	44(42.3)	0.278

n* number of positive cases and positive rate

In male patients, TG elevation was more frequent in distal gastric cancer, N0 stage and I + II stage. TC elevation was more common in early T stage, N stage and TNM stage. Decreased HDL-C was more common in distal gastric cancer, low- and un-differentiated gastric cancer. Elevated LDL-C was common in patients at T1 + T2 stage (Table 4).

In female patients, TC increase was more common in patients with distal gastric cancer(*P* = 0.001) and gastric cancer at early T stage(*P* < 0.001), Table 4.

### Serum lipid level according to TNM stage of gastric cancer

In all patients, Serum TC was higher in early T stage (*t* = 5.667, *P* = 0.000); Serum TG and TC were higher in N0 stage patients (*t* = 3.444, *P* = 0.001, *t* = 3.101, *P* = 0.002) and I + II stage patients (*t* = 3.513, *P <* 0.001, *t* = 4.653, *P* < 0.001). In male patients, TC was higher in T1 + T2 stage (*t* = 5.367, *P <* 0.001); Serum TG and TC were higher in N0 stage patients (*t* = 3.298, *P* = 0.001, *t* = 3.562, *P <* 0.001) and I + II stage patients (*t* = 3.468, *P =* 0.001; *t* = 4.277, *P* < 0.001). In female patients, Serum TC was higher in T1 + T2 stage (*t* = 2.086, *P* = 0.038, Fig. 1).

## Discussion

Dyslipidemia is associated with various chronic diseases, including cancers. The association between dyslipidemia and cancer was first reported in 1909, which found that the presence of “fatty crystals” prevented the tumor from being immobilized by alcohol during the biopsy. In recent years, more and more studies have shown that dyslipidemia is a factor inducing many kinds of malignant diseases. Wei et al. [7] showed that dyslipidemia was related to the occurrence of breast cancer, and lower HDL-C and higher TG levels can promote the progression of breast cancer. Choi et al. [8] reported that low levels of HDL-C increase the risk of colorectal cancer.

In this study, TG, TC and LDL-C levels of gastric cancer patients were higher than those of the control group, while HDL-C levels were lower. In Logistic regression analysis, HDL-C and LDL-C were significantly correlated with the risk of gastric cancer. Zaleska et al. [9] and Hager et al. [10] had reported that the use of many obesity-related drugs [lipid-lowering (statins) or anti-diabetes (metformin)] could reduce the risk of cancer. Campos et al. [11] demonstrated that as one of the intracellular lipid binding proteins (iLBPs), the fatty acid binding protein 5(FABP5) is involved in regulating the uptake, transport and metabolism of fatty acids. It is highly expressed in a variety of malignancies and has been proved to be an oncogene promoting the occurrence and development of cancer. Dong [12] and Bibi et al. [13]reported that transcription factor STAT5A is the main molecular mechanism involved in FABP5 promoting cancer.

The correlation between serum TG levels and gastric cancer was inconsistent in previous literatures. Lindkvist et al. [14] illustrated that when TG was used as a continuous variable, its increase was observed to be significantly correlated with the incidence of gastric cancer. Kim et al. [15] found that higher TG levels were associated with an increased risk of gastric cancer. In our study, although the TG levels in gastric cancer patients were higher than those in the control, the TG level of gastric patients gradually decreased as the progress of the disease, which may be related to the poor intake of patients with gastric cancer, the increase of tumor consumption and the continuous decline of nutritional status.

In this study, there was no significant difference in TC level between gastric cancer patients and healthy control group. But the TC levels of gastric cancer patients with T, N and TNM stages were reduced. A case-control study of Kang et al. [16] found that low TC and HDL-C levels were associated with gastric cancer. They suggested that abnormalities in the apolipoprotein 2 allele were involved in the regulation of TC levels. Matsubara et al. [17] demonstrated that preoperative TC-lymphocyte score was often associated with poor prognosis, which could be used as a new immune-nutritional predictor for the survival of gastric cancer.

Nam et al. [18] from the national cancer center in South Korea showed that low serum HDL-C levels increased the risk of gastric cancer by 2.67 times. Lindkvist et al. [14] also showed that lower HDL-C levels were associated with an increased risk of gastric cancer. Soran et al. [19] showed that the antioxidant properties of HDL-C had a protective effect on tumor progression. Oxidants can damage the DNA of normal cells and promote the transformation into cancer cells. The main mechanism of HDL-C metabolism is to remove harmful oxidants, which can reduce cell damage and prevent cell cancerization. Xu et al’ s meta-analysis found that serum TC and HDL-C levels were inversely correlated with the risk of gastric cancer [20]. Our study showed that HDL-C levels were significantly lower than those of healthy control, which was an important risk factor for gastric cancer. HDL-C levels in patients with gastric cancer were associated with poorly differentiated, low and undifferentiated pathological types.

Limitations and recommendations: This study performed external quality control (EQA) only twice/year. Although, our specimen was cross-sectional case with randomized control from one center to minimize sampling bias, there are still confounding factors due to retrospective analysis. While the eating habits and exercise were important factor for both lipid and gastric cancer. Nevertheless, all these factors were not able to be adjust for, which was limitation in gastric cancer risk estimation. So, a multicenter, large sample prospective study including more influencing factors will be in demand.

HP was classified as group I Carcinogenic risk factor to humans, especially for non-cardiac gastric cancer. In Nam et al. [18] study, HP infection was associated with higher BMI, high serum levels of TC, TG, LDL, fasting plasma glucose, low serum HDL-C levels, which were similar to those in a previous Korean study [21]. The metabolic changes in HP infected subjects may affect gastric cancer risk. In this study, abnormal lipids of TG, TC and HDL-C are more common in distal gastric cancer. It may be associated with HP infection due to more common in distal gastric cancer. However, in present, as well as many of the previous studies on serum lipid and gastric cancer risk, HP infection was not able to be adjust for, which was another limitation in gastric cancer risk estimation.

In conclusion, serum TG, TC and LDL-C levels in gastric cancer patients were higher than health control, while HDL-C levels were relatively lower. HDL-C and LDL-C were significantly correlated with the risk of gastric cancer. But as the progresses of gastric cancer, poor patient intake, increased tumor consumption, and continuous declining in nutritional status, the levels of TC and TG gradually decreased in advanced gastric cancer.

Therefore, high lipid levels are a risk factor for gastric cancer. The daily monitoring of lipid levels and the prevention and management of hyperlipidemia maybe beneficial to reduce the incidence of gastric cancer.

## Data Availability

The datasets used during the current study are available from the corresponding author on reasonable request.

## Abbreviations

FABP5: fatty acid binding protein
iLBPs: intracellular lipid binding proteins
EQA: External quality assessments
HP: Helicobacter pylori
HDL-C: High-density lipoprotein cholesterol
LDL-C: Low-density lipoprotein cholesterol
TG: Triglyceride
TC: Total cholesterol
